# What Is a Biosensor?—A Terminological Guide From Biomolecular Recognition to Bioindicators

**DOI:** 10.1002/elsc.70068

**Published:** 2026-02-04

**Authors:** Tim E. Weber, Anna Fritschen, Menno W. J. Prins, Andreas Blaeser

**Affiliations:** ^1^ BioMedical Printing Technology Technical University of Darmstadt Darmstadt Germany; ^2^ Department of Biomedical Engineering Department of Applied Physics and Science Education Institute For Complex Molecular Systems (ICMS) Eindhoven University of Technology Eindhoven Netherlands; ^3^ Centre For Synthetic Biology Technical University of Darmstadt Darmstadt Germany

**Keywords:** biomolecular sensor, biosensor, chemical biosensor, classification, physical biosensor

## Abstract

Biosensors are an integral part of modern medicine, are used in basic research, and are increasingly used by consumers as point‐of‐care and wearable devices. Meanwhile, the underlying technological approaches are rapidly expanding, including spectroscopic sensing, artificial bioreceptors, synthetic biological approaches, whole‐cell biosensors, and artificial intelligence. With these diversifications in applications as well as technology, the scope and meaning of the term biosensor is blurring. This paper attempts to give an overview of the sensing approaches, with their physical, chemical, biochemical, and biological principles, and an overview of the fields of application, including nonliving systems and living systems. This leads to a comprehensive overview and a reappreciation of the term biosensor, including not only devices with a molecular biorecognition element and physico‐chemical readout but also the sensing of living biological systems using physical and chemical methods, and the use of living biological systems for sensing purposes.

## 1 Introduction

The desire to obtain information about biological systems has accompanied humankind for long. According to records, ancient Egyptians already used simple biological tests to make medical statements [1, 2, 3]. The advent of electrical engineering led to further developments in the field. In 1956, Leland C. Clark Jr. developed a device for his heart‐lung machine to measure the oxygen concentration in a patient's blood in real time [4, 5]. During the calibration process, for which he used glucose and glucose oxidase to generate an oxygen‐free solution, he noticed that his invention could also be used the other way around [4]. By immobilizing the enzyme on an electrode surface and measuring the concentration of oxygen, the biocatalytic reaction could be monitored [4, 6]. With the publication by Clark and Lyons on enzyme electrodes and the opportunity to detect biomolecular analytes, Clark became known as one of the fathers of the field of biosensing [4, 7].

Through the work of many scientists over the past decades, new recognition methods and transducer principles were developed, and new types of biosensors appeared. Prominent examples are Guilbault and Montalvo, who invented a potentiometric biosensor to detect urea, and Suzuki et al. and their discovery of a microbe‐based immunosensor and many more, as described in the literature [6, 8, 9, 10]. The first successful commercialization was accomplished by Yellow Springs Instruments (YSI Inc.) with their *Model 23 Glucose Analyzer* product in 1975 [6, 11]. Terms such as *enzyme electrode* and *ion selective electrode* were mostly used, although *biosensor* already existed as a sensor class description [12]. In 1987, Turner described that these devices consist of a biological sensing element connected to or integrated into a transducer. This definition has been widely adopted in literature and is the foundation of the International Union of Pure and Applied Chemistry (IUPAC) definition for electrochemical biosensors formulated in 1992 [13, 14].

Today, biosensors are an essential part of our society with many different fields of applications, utilizing advancements from different research fields to improve functionality and application possibilities. Advances in biomolecular engineering and in (nano)materials such as gold nanoparticles, graphene, and hybrid nanomaterials have improved the sensitivity and selectivity of biosensors and enabled applications in more complex matrices [15, 16, 17, 18]. Biological recognition methods based on enzymes and antibodies, and amplification methods have allowed the detection of minimal biomarker concentrations, enabling high precision and fast diagnostics [19]. Biosensors are used in environmental monitoring, for example, in water quality and soil analysis, air pollution monitoring, and biodiversity preservation, aiming to mitigate the effects of climate change, environmental pollution, and mismanagement of resources [20]. Antibiotic residuals, pathogens, and pollutants, such as heavy metals, pesticides, microbial contaminants, and greenhouse gases, are prominent analytes of interest [15, 20]. Biosensors are used throughout the food supply chain, from the pre‐harvest phase to inline quality control in industrial processes and smart food packaging, enabling customers to evaluate food quality [21, 22, 23, 24, 25]. Antibiotic residues, pathogens, fungi and, above all, food contamination are of interest here [15, 23]. In the medical sector, point‐of‐care testing and wearable biosensors enable patient monitoring and testing without the need for a doctor [18, 26, 27, 28, 29]. Biosensors support drug development and implementations in Organ‐on‐a‐Chip‐Systems support bridging the gap between animal and human trials [30, 31]. Methods such as machine learning (ML) and artificial intelligence (AI) are used for data processing with the hope of supporting disease diagnostics through pattern recognition and potentially finding unknown biomarkers or combinations, for example [29, 32, 33]. Nowadays, space research is an emerging field in which biosensors are being used [34, 35, 36]. Due to their small size and weight, they are a cost‐effective payload in nanosatellites [36]. This allows, for example, the effects of cosmic radiation on organisms such as yeast to be investigated to better understand its effects on astronauts and thus support future deep space missions [34, 36]. The utilization of novel materials and detection methods, as well the inclusion of modern research approaches such as AI or ML, enable biosensor technology to continuously improve and be widely applied in society [32, 33].

The wide use of biosensor technology, from basic research to many commercial products, causes the term “biosensor” to be used with different meanings, depending on the technologies, the applications, and the backgrounds of the people involved. In an attempt to facilitate communication across disciplines and to help people familiarize with the different concepts, we give an overview of sensing approaches, with their physical, chemical, biochemical, and biological principles, and an overview of fields of application, including nonliving systems and living systems. This leads to a comprehensive overview and a reappreciation of the term biosensor, including not only devices with a molecular biorecognition element and physico‐chemical readout but also the sensing of living biological systems using physical and chemical methods, and the use of living biological systems for sensing purposes.

## 2 What Is a Sensor and How Does a Biosensor Fit in?

**FIGURE 1:**
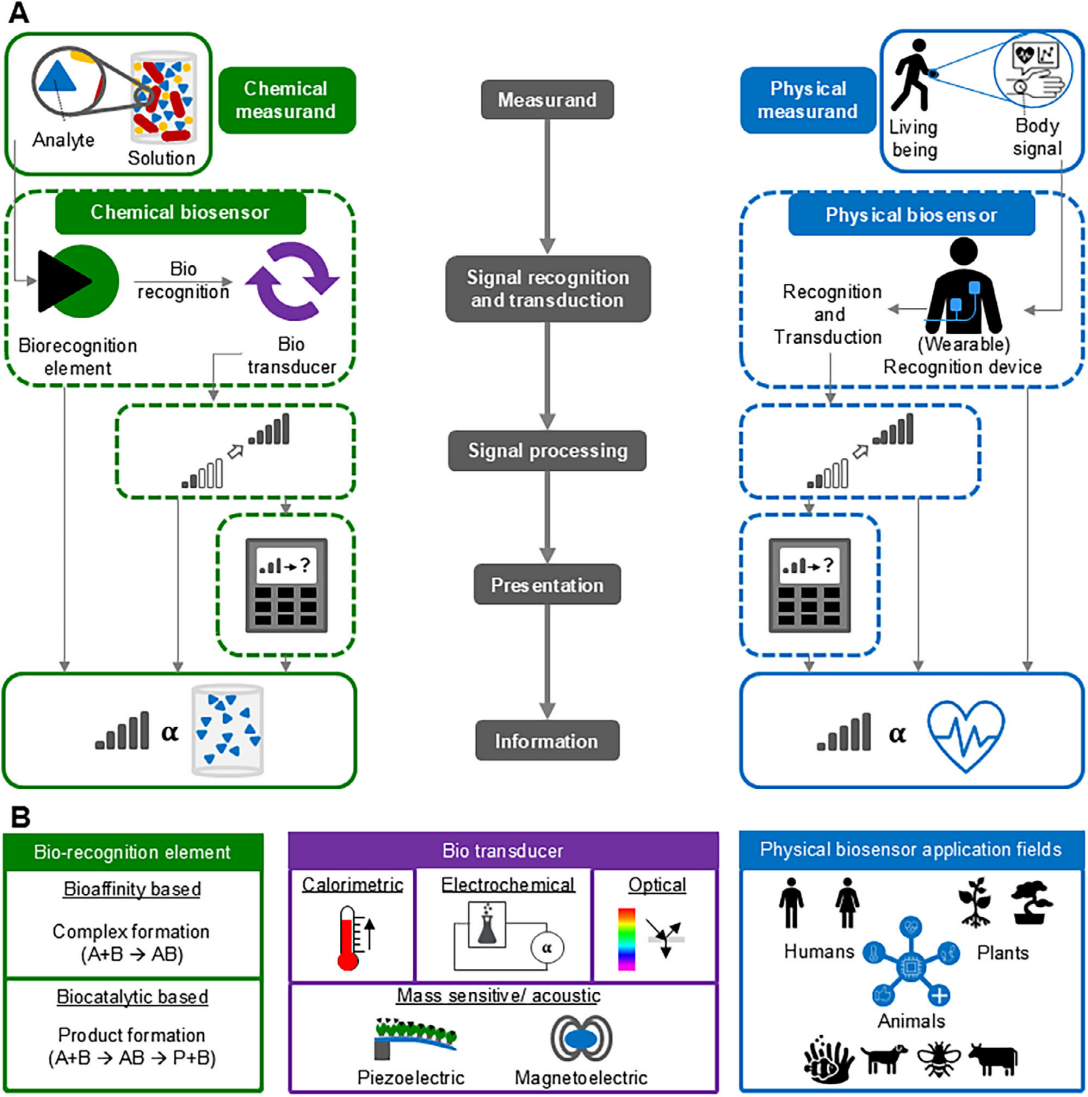
Schematic illustration of chemical and physical biosensors. (A) Structure and working principles of chemical biosensors (left) and physical biosensors (right). (B) Biorecognition principles [49] and transducer principles [50] for chemical biosensors as well as living biological systems as application fields of physical biosensors.

A sensor is a device that is able to detect changes in real‐world parameter, usually called a measurand (Figure 1A). The process is called *measuring* or *sensing*. The measured value is converted into a usable signal that is either presented to a user (e.g., a number) or used as input for a regulating process, for example, a feedback process. A sensor consists of at least two components. One part that changes its properties in response to an environmental change (*recognition*) and another part that converts the change into a usable signal (*transduction*) [37]. For a mercury‐in‐glass temperature sensor, the property‐changing component is the mercury, and the converting component is the tube‐shaped glass with a scale. In addition to the presentation of the signal, signal processing might also be a further necessary component of a sensor. Terms such as *sensor*, *measuring device*, and *sensor system* are often used synonymously, without a clear definition of their components. This results in a variety of different approaches to the classification of sensors. The definitions depend, for example, on the application, for example, direct or indirect measurement of the measurand, as is discussed in publications and books [8, 37, 38].

*Physical sensors* are described as devices measuring physical quantities such as pressure, temperature, or electrical voltage and transforming them into usable information [38]. Analog to this, *chemical sensors* measure chemical quantities such as pH value, a specific *analyte* presence as well as its concentration, or a composition of a solution [14, 37]. Following that approach, a biosensor, short for *biological sensor*, is a sensor that captures information about the state of a biological system and thus measuring biological quantities such as heart rate or blood glucose level. However, in some literature biosensors are viewed more narrowly as a subclass of chemical sensors [14, 37].

## 3 Biosensors—A Subclass of Chemical Sensors

The IUPAC has defined biosensors in the following way: *“Biosensors are chemical sensors in which the recognition system utilizes a biochemical mechanism […]”* [14]. This definition stems from the field of electrochemical biosensors, where biosensors are viewed as a subclass of chemical sensors [14]. The crucial difference to other chemical sensors is the receptor or recognition element. This consists of a biological component and is therefore often referred to as a *biorecognition element* or *bioreceptor*. According to this definition, a biosensor consists of at least two components: a bioreceptor (biorecognition element) and a (bio)transducer (Figure 1A). The transducer converts the biological/chemical reaction between the target analyte and the bioreceptor into a physico‐chemical readout. In analogy to the general concept of sensors, further components can be added, such as signal processing units and a display.

**FIGURE 2:**
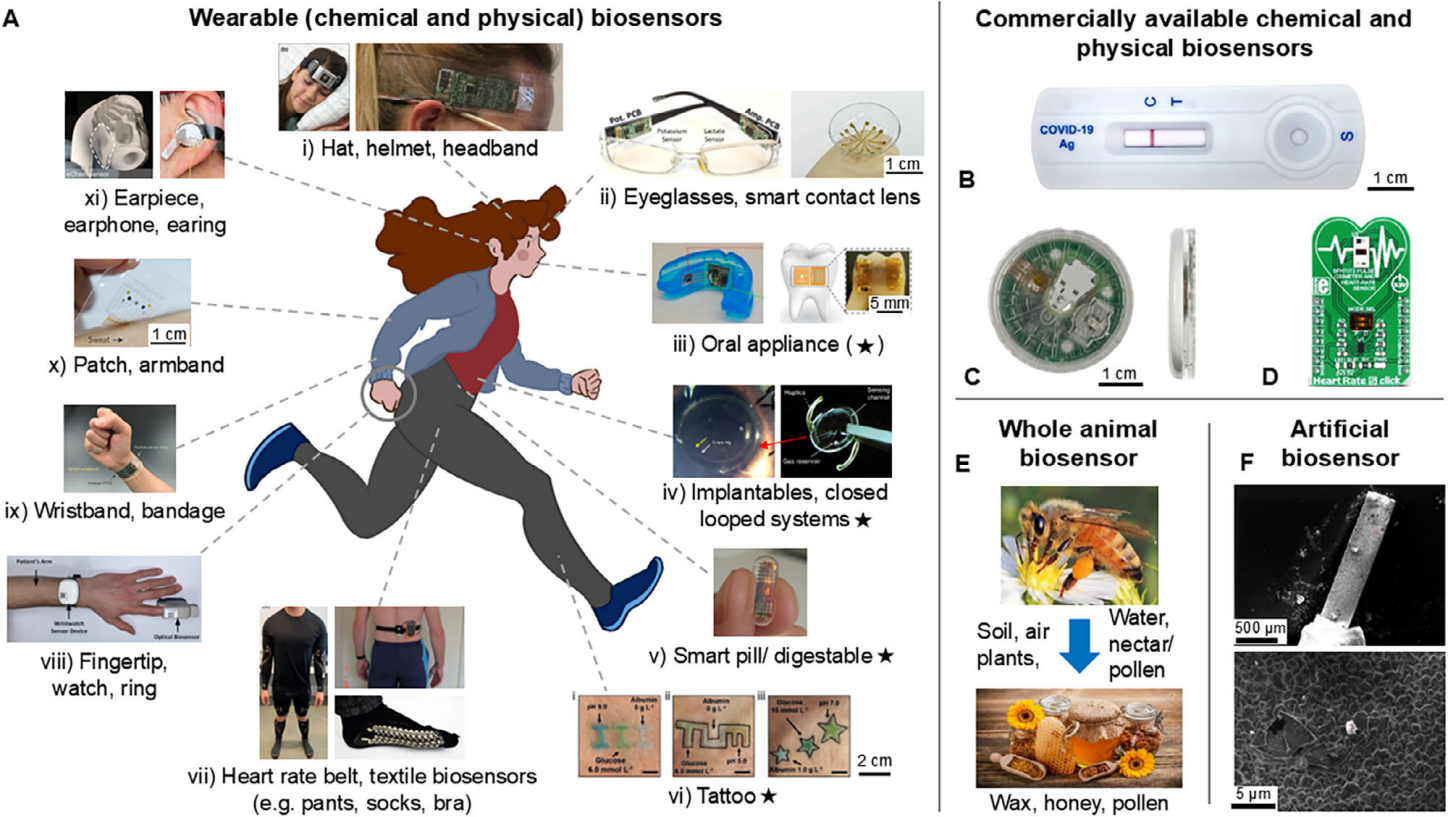
Examples of physical and chemical biosensors in research and commercial applications. (A) Examples of wearable biosensors and often relatively smaller inversive biosensors (marked with a star symbol): (i) Head worn biosensors for sleep or perspiration monitoring (Reprinted from [60], Copyright 2016 European Sleep Research Society, reproduced with permission from John Wiley and Sons) and (Reprinted from [44], reproduced with permission from SNCSC); (ii) Optical devices, for example, for measuring glucose, electrolytes, or sepsis marker such as TNF‐α (Used with permission of Royal Society of Chemistry [61], permission conveyed through Copyright Clearance Center, Inc.) and (Reprinted from [62], Copyright 2019 Wiley‐VCH Verlag GmbH & Co. KGaA, Weinheim, reproduced with permission from John Wiley and Sons); (iii) Salivary biosensors for uric acid and bacteria monitoring (Reprinted from [63], Copyright (2015), with permission from Elsevier) and (Reprinted from [64], reproduced with permission from SNCSC); (iv) Implantable biosensors and closed‐loop systems, here for in vivo interocular pressure monitoring (Reprinted from [65], reproduced with permission from SNCSC); (v) Digestible smart pill (Reprinted from [66], Copyright (2021), with permission from Elsevier); (vi) Colorimetric dermal tattoo biosensors for metabolite monitoring (Reprinted from [67], Copyright 2019 Wiley‐VCH Verlag GmbH & Co. KGaA, Weinheim, with permission from John Wiley and Sons); (vii) Body‐worn biosensor systems for activity or sweat monitoring (Used with permission of Royal Society of Chemistry, from [68], permission conveyed through Copyright Clearance Center, Inc.) and (Used with permission of Royal Society of Chemistry, from [69], permission conveyed through Copyright Clearance Center, Inc.) and (Reprinted from [70], Copyright 2022, This is a U.S. Government work and not under copyright protection in the US; foreign copyright protection may apply, reproduced with permission under a Creative Commons Attribution (CC‐BY 4.0) License from Springer Nature, https://doi.org/10.1038/s41598‐022‐13701‐4); (viii) Finger and watch optical based biosensors for oxygen saturation and heart rate measurement (Reprinted from [71], Copyright 2020 by the authors, reproduced with permission under a Creative Commons Attribution (CC‐BY 4.0) License from MDPI, https://doi.org/10.3390/s20061675); (ix) Wristband biosensors for in situ perspiration analysis (Reprinted from [44], reproduced with permission from SNCSC); (x) Patch based electrochemical biosensors for sweat analysis (Reprinted from [73], reproduced with permission from SNCSC); (xi) Electrochemical and ‐physiological biosensor system for monitoring sweat and brain activity (Reprinted and adapted from [74], Copyright 2023, The Author(s), reproduced with permission under a Creative Commons Attribution (CC‐BY 4.0) License from Springer Nature, https://doi.org/10.1038/s41551‐023‐01095‐1). (B) Commercially available lateral flow test for SARS‐CoV‐2 antigen detection (photo taken by the authors). (C) Commercially available glucose meter (photos taken by the authors). (D) Physical sensor platform for heart rate monitoring (Reprinted from https://www.mikroe.com/heart‐rate‐5‐click with permission from MikroElectronika d.o.o.) [75]. (E) Use of bees as biosensors for environmental monitoring (Reprinted and adapted from [76], Copyright 2020, The Authors, reproduced with permission under a Creative Commons Attribution (CC‐BY 4.0) License from Springer Nature, https://doi.org/10.1007/s41207‐020‐00204‐9). (F) SEM image of a molecularly imprinted polymer (MIP) (Reprinted and adapted from [77], Copyright 2024, The Authors, reproduced with permission under a Creative Commons Attribution (CC‐BY 4.0) License from American Chemical Society, https://doi.org/10.1021/acsomega.4c02906).

Biosensors that contain a bioreceptor as well as a transducer to monitor a chemical measurand are referred to as *chemical biosensors* or *molecular (bio)sensors*. These terms offer a more specific description of a biosensor system [39, 40]. Chemical biosensors can be applied to acquire information from both living and nonliving systems. For example, to measure analytes linked to diseases such as cancer or bacteria detection for the monitoring of food contaminations, or to measure heavy metals like mercury or lead in a nonliving system such as water [23, 41, 42, 43]. The sensing devices can be wearable devices (e.g., for lactate or glucose monitoring in sweat, or via microneedles in interstitial fluid) or point‐of‐care solutions, such as the well‐known paper‐based lateral flow tests for COVID‐19, for pregnancy hormone, or for antibiotics (Figure 2A–C) [44, 45, 46, 47, 48].

Chemical biosensors can be classified in the same way as sensors in general, for example, into active and passive or direct and indirect systems, but there are also field specific approaches [8, 51]. Besides the classification based on the detection system or the technology, chemical biosensor classifications are based most commonly on the two main components [8]. For the bioreceptor, many different biological materials can be used, such as enzymes, aptamers, or antibodies [50]. Through this, chemical biosensors can, for example, be distinguished into *bioaffinity* or *biocomplexing‐based biosensors*, when utilizing components for the bioreceptor such as antibodies, other proteins or aptamers, or into *metabolism* or *biocatalytic‐based biosensors* when using enzymes or cells as bioreceptors (Figure 1B) [14, 49, 52]. Another approach is the classification into specific classes, such as DNA, enzyme, or phage biosensor, for example [53]. Chemical biosensors are sometimes also classified directly by their bioreceptor type, such as aptamer‐based biosensors. To emphasize this aspect, abbreviations, such as *aptasensor*, which refers to biosensors that work with aptamers, or *immunosensor*, which is based on antigen–antibody interaction, are routinely used [54, 55]. Analogously, the transducer principle can be highlighted by using terms such as electrochemical, thermal/calorimetric, acoustic (piezoelectric), or optical biosensor (Figure 1B)  [8, 53]. In the literature, these terms are also used in compound form, for example, “[…] *electrochemical affinity‐based biosensor* […]” [31]. An understanding of the terminological description and its semantic breakdown is important in order to understand and communicate the basic functionalities of a biosensor.

## 4 Biosensors for Measuring Physical Signals

With the definition of biosensors as a subclass of chemical sensors by the IUPAC, as described in the previous section, the question arises whether such a subclass also exists for physical sensors. Since a large part of the scientific community in the field of biosensors shares the perspective of biosensors as a subclass of chemical sensors, there are only a few publications that describe a different perspective. Here, biosensors either measure exclusively physical signals or add these to chemically derived measurements (Figure 1A). These can be physiological signals, vital signs, mobility, number of steps, calories burned, pulse, respiratory rate, intraocular pressure (IOP), blood, or body temperature, as examples (Figure 2A). An example of such a broader approach is the description for biosensors given by Yang et al.: “*Biosensors refer to the devices that record the information of life process by capturing biomarkers including IOP, heart rate, metabolites, bacteria, and hormones to offer valuable insights into the health of persons”* [28]. In literature, often explicit reference is made to what is measured, for example, *“[…] physiological textile biosensors for registering different vital signs of the body”* [56]. Vital signs are often measured in a wearable biosensor format [57, 58]. The appearance and measurement task (chemical and/or physical) of wearable biosensors can vary greatly (Figure 2A). The measured variables do not necessarily have to provide information about the state of health of a biological being but can also be measured for other purposes. For example, Zhang and coworkers track the eye movements of a wheelchair user with the help of a so called hydrogel biosensor via electrooculogram and strain signals to enable steering of a wheelchair [59].

In conclusion, employing a physical measurement mechanism, to study a biological or living system (humans, plants, or animals) is a common theme in biosensor literature and these systems are further referred to as *physical biosensors* (Figure 1B). This contrasts their usage to the previously described chemical biosensors, which are also used for sensing nonliving systems. Sometimes the term *physiological biosensor* is used [78]. To describe both chemical biosensors and physical biosensors for vital signs or *behavioral data acquisition*, in some cases the prefix “bio” is even omitted to rather emphasize other aspects of the sensor, for example, *medical sensors*, *biometric monitoring devices*, *wearable (bio)sensors*, or *wearable hybrid sensing systems* [6, 79, 80].

## 5 Commercial Biosensors and Consumer Understanding

Since the launch of the first commercial biosensor *Model 23 Glucose Analyzer* in 1975 by the company YSI, the technology of biosensors has been used in a broad field of applications [6, 11]. This includes not only environmental safety, medical diagnostics, and drug development but also application areas such as security and safety applications, or space research [30, 35, 50, 81, 82, 83]. For medical applications, many commercial solutions are already available, used by both medical institutions and consumers. The most prominent examples are lateral flow tests, such as pregnancy and COVID‐19 rapid tests, and the variety of electrochemical glucose meters for patients with diabetes (Figure 2B, C).

The sale of commercially available products is accompanied by advertising. Health trackers, smart watches and fitness gadgets (wearable biosensors) or developing platforms for these (Figure 2D), are mentioned in advertisements and expose the average consumer to the term biosensor as a device that measures quantities such as steps, heart rate, or other vital signs. Both small companies and large corporations use the term biosensor in their advertising. Consequently, consumers may tend to think of biosensors as physical biosensors, while scientists may tend to think of biosensors as chemical biosensors.

## 6 Bioindicators and Biosensors With Synthetic Biorecognition Elements

As a next step, beyond physical biosensors and chemical biosensors, biosensors can have a living biological system as recognition and transduction principles. The living biological system can be a whole cell, a tissue, or even a whole organism such as a plant or an animal (e.g., a bee, bird, mussels, or dog), or a part thereof, such as the antennae of a silk moth, for example [84]. A drug‐sniffing dog at the airport is an animal that fulfills a specific chemical measuring task (qualitative) by use of its nose (olfactory biorecognition element) and by providing an output signal (barking) that is understandable for a human. It therefore fulfills the requirements of the IUPAC definition for chemical biosensors presented above. In the case of environmental monitoring, living sensor systems are referred to as *bioindicators* (qualitative) or *biomonitors* (quantitative) [85]. Here, an organism (animal/plant) or community indicates a system condition through its response (e.g., physiological, chemical, or behavioral) and shows changes or biotic responses to environmental stress [85, 86]. Examples for this are bees that collect pollen and whose honey quality is used to monitor air pollution, or changes of shellfish behavior to monitor water quality (Figure 2E) [87, 88, 89]. Although not recommended by the IUPAC, the term bioindicator is sometimes also used to describe disposable biosensor systems [14]. There are also approaches that use plants or parts thereof as recognition elements [90], called as *cyborg botany*, *electronic plants*, or bioindicators [86, 91, 92]. As these systems consist of a biorecognition element (here whole or parts of plants), coupled to a transducer, they meet the requirements of the definition of a chemical biosensor.

A further complication is that biorecognition elements of chemical biosensors can be synthetic rather than biological. The biorecognition elements can be aptamers, which belong to the field of synthetic biology or molecular imprinted polymers (MIPs), for example [93]. For the latter, a polymer is molecularly imprinted with the desired target analyte and subsequent removal creates a polymer with analyte‐specific three‐dimensional cavities that mimic the function of natural biorecognition element (Figure 2F) [50, 77]. In the literature, MIPs are already used as a biorecognition elements for biosensors and thus represent a good example of an *artificial biorecognition element* in an *artificial biosensor* [50, 93, 94].

## 7 Challenges and Futures

Biosensors have been under development for nearly 70 years with groundbreaking contributions and innovations. The large number of possible bioreceptors and transducers require a wide range of fabrication and processing methods. Biological components often need to be processed gently, for example, using sputtering or printing technologies such as inkjet printing. For technical components, classical methods are employed, such as vapor deposition and lithography from microelectronics. The integration of biological components into technical subsystems creates particular complexity. On the one hand, many different backgrounds and close interdisciplinary cooperation is needed to address this challenge; on the other hand, the biological components may exhibit alternative behavior in a different environment, making predictions more difficult during development [18]. As a result, biosensor systems almost always need to be developed individually and fabricated under special conditions.

Additionally in case of a suitable target group, successful commercialization of a biosensor requires addressing further challenging obstacles. Shelf life requirements, often limited by the stability of biological components and complex regulatory requirements in case of medical products, lead to long development phases and increase the difficulty to map development and production costs [18, 95]. Only a fraction of the existing proof‐of‐concept‐technologies is successfully transferred into commercially available products [95]. Many proofs‐of‐concepts are not transferred to the commercial sector, but they clearly demonstrate the development of biosensor technology today. Biosensor key characteristics, selectivity and sensitivity, are often challenged when it comes to complex matrices such as in environmental monitoring or diagnostics (e.g., wastewater or full blood sample), limiting their competitiveness compared to existing laboratory methods [18]. Without sample preparation, components similar to the target analyte can cause signal interferences that hinder the detection of low concentrations in real samples [18]. Researchers are studying new materials such as carbon nanotubes, graphene or gold nanoparticles, and new amplification methods such as CRISPR/Cas, for example, to improve the sensitivity and selectivity and thus increase the competitiveness compared to existing laboratory methods, potentially supporting commercialization in the future [15, 17, 19, 62, 96].

Recent trends show a shift from purely analog biosensor systems toward *smart biosensors* that incorporate advanced computation‐aided signal processing. Decentralization into resource limited settings may be enabled by technologies such as Internet of Things (IoT) [33]. Emerging approaches incorporate ML and AI into biosensor systems [32, 33]. This may improve the data processing and enable complex interpretation approaches such as pattern recognition for real‐time decision‐making and personalized diagnostics [32]. Supervised learning yields promising opportunities for predictive models, for example, disease detection, whereas unsupervised approaches could uncover new unknown biomarkers or biomarker patterns for diseases [29, 32, 33]. To fully incorporate such digital technologies in biosensors several new questions need to be answered, such as concerns regarding data privacy, security, or regulations, for example [32, 33]. Overall, the emerging materials and novel biorecognition methods mentioned above, as well as modern digital approaches such as AI and ML, can further improve the sensitivity, adaptability, and robustness of biosensors, leading toward a new generation of biosensors.

## 8 Summary and Conclusion

Biosensors encompass a plethora of mechanisms of action, ranging from chemical and physical measurement concepts to the use of tissues or animals that serve as bioindicators, as well as a wide diversity of applications, including living and nonliving systems. In addition, there are often prefixes, convoluted word constructions (e.g., electrochemical biosensor) or abbreviations (e.g., aptasensor) that refer to certain properties of the biosensor. If the commercial uses of the word biosensors are included, then the term is further expanded.

**FIGURE 3:**
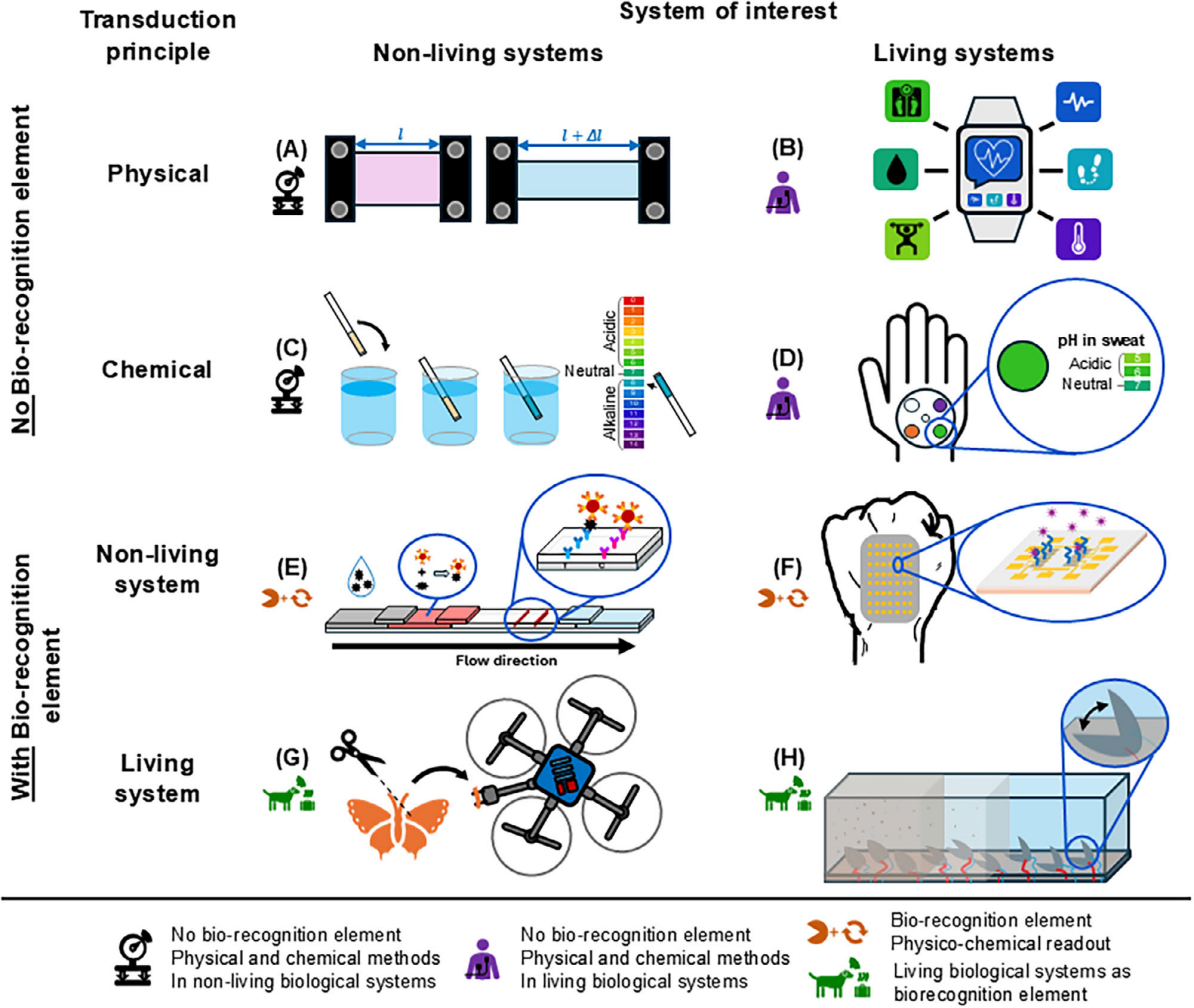
Examples for different (bio)sensor systems as classified in Table 1. (A) Colorimetric strain gauge [97]; (B) Smart watch for monitoring different vital signs; (C) pH test stripes; (D) Epidermal microfluidic sweat sensor on skin for pH monitoring [72]; (E) Antibody based lateral flow test; (F) Field effect transistor skin patch with aptamers as bioreceptors [62]; (G) Biosensor drone with an integrated silk moth antenna as the bioreceptor [84]; and (H) Bivalves for marine monitoring via valve‐gaping [89].

The collected perspectives can be summarized in an overview of the sensing approaches, with their physical, chemical, biochemical, and biological principles; and an overview of the fields of application, including nonliving systems and living systems (Table 1, Figure 3). The intersections related to biosensors are indicated in colors: purple, orange, and green. Purple refers to living biological systems that are being sensed using physical and chemical methods, without using biorecognition elements. Orange refers to sensors based on biorecognition elements and physico‐chemical readout, which fits the IUPAC definition of a chemical biosensor. Green refers to living biological systems that are used for sensing purposes.

**TABLE 1:** Classification of sensing technologies, based on technological approaches and applications.

		Biorecognition element	Transduction principle	System of interest			
				Nonliving systems e.g., *fluid with molecular components, chemical substance, technical components, machines*		Living systems e.g., *cells, organ, organism, ecosystem, human, body fluids*	
Front‐end measurement principle	Physical	No	Physical	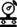	Sensors that measure physical quantities, applied to nonliving systems e.g., *strain gauges* [97], *pressure sensors* [98], *temperature sensors* [99]	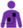	Sensors that measure physical quantities, applied to living systems e.g., *physiological signals, vital signs, mobility, such as cerebral hemodynamic monitoring* [100]*, intraocular pressure* [101]*, smart scales for weight measurement and body composition* [102]
	Chemical	No	Chemical	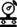	Sensors that measure chemical properties, using chemical transduction principles, applied to nonliving systems e.g., *pH* *indicators* [103], *chemical assays* [103], *redox reactions* [104]	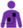	Sensors that measure chemical properties, using chemical transduction principles, applied to living systems e.g., *chemical compounds in sweat* [72]*, chemical environmental pollution* [105]
		Yes e.g., antibody, aptamer, enzyme, MIP	Nonliving system physico‐chemical readout	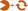	Sensors that use a molecular biorecognition element, applied to nonliving systems e.g., *enzymatic electrochemical biosensors for ethanol detection in beer* [106], *immunosensor for continuous protein monitoring in a purification process* [107]	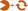	Sensors that use a molecular biorecognition element, applied to living systems e.g., *glucose sensors for patients with diabetes, biosensors for infection detection* [108]*, environmental monitoring* [47]*, continuous monitoring in an organ‐on‐a‐chip‐device* [31]
		Yes e.g., cell, organism, tissue, whole animal, or plant	Living system reaction of the living system	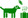	Biosensors with a living system as a transduction principle, applied to nonliving systems e.g., *whole cell biosensors for detection of explosives and heavy metals* [109], *cell biosensors used in deep space research* [34, 36], *cell biosensors in industrial processes* [22, 24, 51]	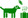	Biosensors with a living system as a transduction principle, applied to living systems e.g., *plants as sensors* [92]*, bees or shellfish for environmental monitoring* [87, 110]
Legend	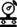		Black: nonliving biological systems are being sensed using physical and chemical methods, without using biorecognition elements.				
	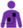		Purple: living biological systems are being sensed using physical and chemical methods, without using biorecognition elements.				
	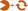		Orange: sensors based on biorecognition elements and physico‐chemical readout.				
	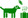		Green: living biological systems are used for sensing purposes.				

Sections related to biosensing and biosensors are indicated in colors: purple, orange, and green.

A chemical biosensor consists of a biorecognition element and a transducer, with synthetic biorecognition elements also being used. Its field of application includes both living system (e.g., a virus for disease detection) and nonliving systems (e.g., metal ions in water) [42, 43, 46]. The reverse applies to physical biosensors. They are applied to living systems and based on the sensing of a wide variety of physical parameters. In addition, certain properties of a biosensor can be emphasized by adding prefixes. The emphasis can be on the type of bioreceptor (e.g., aptamer‐based biosensor), the mechanism of action of the transducer (optical biosensor), the field of application (e.g., medical biosensor), or its device format (e.g., wearable biosensor).

In summary, the different uses of the term biosensor originate from the rapid development and diversity of the underlying technological approaches and the wide range of applications that are being served. The biosensing field comprises not only devices with a molecular biorecognition element and physico‐chemical readout but also the sensing of living biological systems using physical and chemical methods, and the use of living biological systems for sensing purposes. There is currently no definition of the term biosensor that encompasses all characteristics, which means that the term relates to the context of use. We recommend that scientists are aware of the diverse perspectives and should clearly define in their communications the meaning and context of their use of the term biosensor.

## Funding

The authors have nothing to report.

## Conflicts of Interest

For literature review, a broad internet search was conducted. Scientific literature on biosensors was examined and compared with the IUPAC definition presented to identify deviating concepts. From these concepts, generalized approaches and limitations were developed through abstraction, and further targeted research was conducted. A general internet search was conducted to research the industry and customer perspective. The focus here was on publicly accessible company websites and advertising material rather than scientific publications. No products or companies were given preference or weighted consideration. The authors declare no conflicts of interest.

## Data Availability

Data sharing is not applicable to this article, as no new data were created or analyzed in this study.
